# Chylous Ascites in Systemic Lupus Erythematosus: A Diagnostic Challenge

**DOI:** 10.7759/cureus.1226

**Published:** 2017-05-05

**Authors:** Badar Hasan, Talal Asif, Maryam Hasan, Amr F Edrees

**Affiliations:** 1 Department of Internal Medicine, University of Missouri Kansas City (UMKC); 2 Internal Medicine, NYU Langone Medical Center, New York; 3 Department of Internal Medicine and Rheumatology, University of Missouri Kansas City (UMKC)

**Keywords:** chylous ascites, sle

## Abstract

Systemic lupus erythematous (SLE) is a chronic inflammatory disease of unknown etiology. It can affect nearly any organ. Gastrointestinal (GI) involvement in SLE is frequent but is mostly related to medication side effects and concomitant infections. Chylous ascites is a rare form of ascites that is milky appearing due to the high concentration of triglycerides. Chylous ascites as a complication of SLE is atypical. Our case highlights an extremely rare presentation of chylous ascites in SLE as an initial manifestation of the disease itself, posing diagnostic and therapeutic challenges. Through this case, we aim to raise awareness of SLE as a rare but reversible cause of chylous ascites.

## Introduction

Systemic lupus erythematous (SLE) is an autoimmune disease affecting multiple organs [1]. Gastrointestinal (GI) involvement is seen in up to 40% of the cases [2]. GI manifestations in SLE are wide-ranging and include gastroesophageal reflux disease, dysphagia, diarrhea, constipation, intestinal obstruction, hemorrhage, perforation, protein-losing enteropathy, and malabsorption [2]. Ascites is very rare is SLE [3]. Ascites in the setting of SLE is seen in the presence of nephritic syndrome, congestive cardiac failure, or hepatic cirrhosis [3]. Chylous ascites is the accumulation of milky appearing triglyceride-rich fluid in the peritoneal cavity [4]. The chylous ascitic fluid is derived from intestinal or thoracic lymphatic vessels [5]. Chylous ascites in association with SLE has only been described in a few case reports and presents a diagnostic challenge especially when this is the initial mode of presentation. Here, we describe a rare case of chylous ascites in a patient eventually diagnosed with SLE. Our case serves to remind practitioners of the protean and diverse manifestations of SLE. Furthermore, through this case, we also advocate consideration of SLE in the differential of any patient with chylous ascites so that this crucial syndrome can be diagnosed in time.

## Case presentation

A 52-year-old African-American female with past medical history significant for essential hypertension presented to us with the chief complaint of severe abdominal distention. The patient reported that she had been experiencing dyspnea on minimal exertion, generalized weakness, and progressive abdominal distention for the past four months. She denied chest pain, orthopnea, paroxysmal nocturnal dyspnea, cough, melena, hematochezia, nausea, vomiting, diarrhea, fever, rash, or joint pains. She also denied any recent travel history, abdominal trauma, or surgery.

On physical examination, her blood pressure was 127/65, pulse 92/minute, temperature 97.8°F, and respiratory rate 14/minute. On abdominal examination, she had a distended abdomen with positive fluid thrill. There was mild diffuse tenderness to deep palpation. Bowel sounds were normal. Rest of the systemic exam showed 2+ bilateral pedal edema, no jugular venous distention, normal cardiovascular and pulmonary examination.

**Figure 1:**
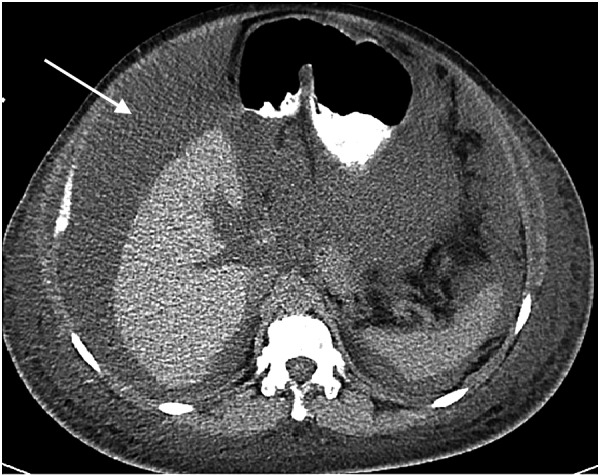
Computed tomographic (CT) scan of the abdomen showing marked amount of abdominal ascites (arrow) with fluid surrounding the spleen and the liver.

Computed tomographic (CT) scan of the abdomen and pelvis was performed first that revealed marked abdominal and pelvic ascites (Figure 1).

Ultrasound of the abdomen indicated normal liver architecture and portal vein size.

**Figure 2:**
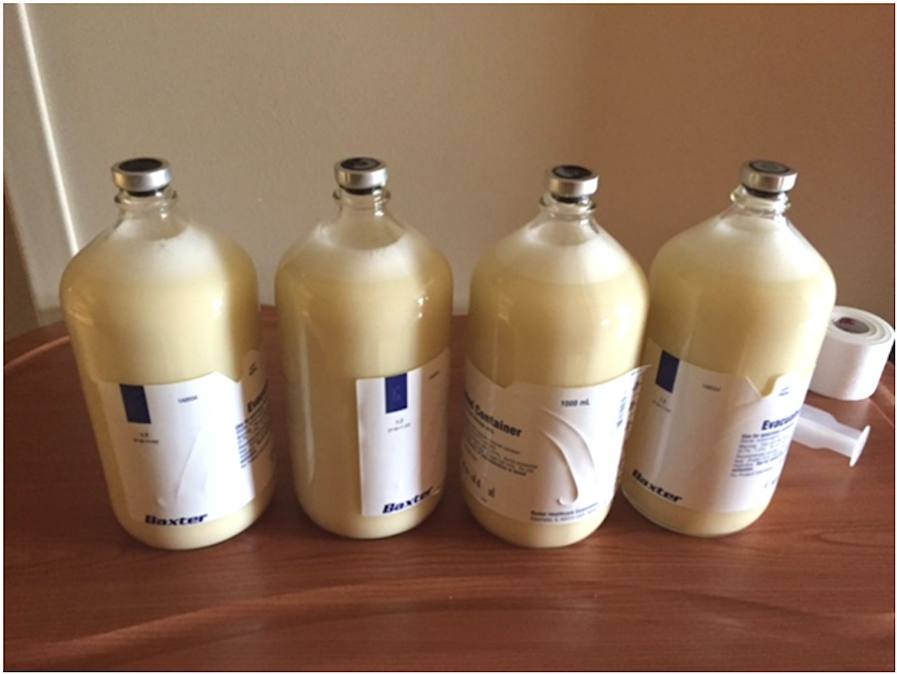
Four liters of milky white appearing peritoneal fluid on paracentesis.

The patient underwent diagnostic and therapeutic paracentesis with drainage of four liters of chylous appearing fluid as shown in Figure 2.

Ascitic fluid analysis showed elevated triglyceride level of 1732 mg/dl, white cell count of 6/cmm, albumin of 1.0 mg/dl, serum–ascites gradient (SAAG) of 0.3, negative gram stain and culture. Cytology of the ascitic fluid revealed mixed inflammatory cells but no atypical cells.

Gastroenterology team consult was requested. The most important approach recommended at this time was to rule out malignancy, nephrotic syndrome, right heart failure, tuberculosis, pancreatitis, and inflammatory causes. Rheumatology team opinion was also obtained. There was low suspicion of cirrhosis on the basis of above mentioned abdominal imaging findings.

Further labs revealed normal white cell count, hemoglobin of 8.2 g/dl which was her baseline with iron studies suggesting anemia of chronic disease, normal platelet count, normal serum lipase, proteinuria of 1 g/day, and elevated serum creatinine of 1.08 mg/dl from a baseline of 0.7 mg/dl. Serological testing showed positive antinuclear antibody (ANA), positive antidouble-stranded deoxyribonucleic acid (anti-dsDNA) antibody, positive anti-Smith antibody (anti-Sm), low C3 and C4 complement levels, and elevated erythrocyte sedimentation rate (ESR) of 118 mm/hr. Liver function tests were normal except for a very low serum albumin level of 1.3 mg/dl. Fasting lipid panel, hepatitis B and C serology, interferon-gamma release assay for tuberculosis (T-spot test), human immunodeficiency virus (HIV) testing, antineutrophil cytoplasmic antibody (ANCA), alpha fetoprotein (AFP), carcinoembryonic antigen (CEA), and urine drug screen were all within normal limits. Transthoracic echocardiogram showed trace pericardial effusion with preserved ejection fraction and normal pulmonary artery pressures.

The patient underwent inguinal lymph node biopsy which was consistent with reactive lymphadenopathy. CT of the chest was performed to rule out underlying malignancy and was unremarkable.

With these clinical findings and laboratory parameters, the patient was diagnosed with SLE. Renal biopsy was pursued due to suspicion of nephritic syndrome which came back positive for class V lupus nephritis.

The patient was treated with intravenous methylprednisolone followed by prednisone taper. Over the course of two months, the patient remained symptomatic requiring multiple therapeutic paracentesis. Initiation of monthly cyclophosphamide infusions leads to resolution of ascites and improvement in her symptoms.

## Discussion

The incidence of chylous ascites has been estimated between 1 in 20,000 to 1 in 187,000 admissions at large tertiary care referral centers [4]. Chylous ascitic fluid is exudative in nature and is rich in triglycerides, chylomicrons, and lymphocytes. Triglyceride levels of greater than 110 mg/dl in the peritoneal fluid supports this diagnosis [6]. Traditionally origin of chylous ascites has been divided into traumatic (including abdominal surgeries) and non-traumatic, both having equal incidence.

The most common causes of chylous ascites in the Western population are abdominal malignancy and cirrhosis while tuberculosis and parasitic infection remain the principal causes in developing countries [7]. The most common clinical presentation remains progressive painless abdominal distention as seen in our case [4]. Chylous ascites in SLE has only been described in rare case reports with our literature review revealing only 12 cases, making this case even more significant due to the dearth of data on this subject matter.

Approach to patients with chylous ascites involves targeting the possible causes. In our case, the patient had no history of trauma or surgery. The clinical, imaging, and lab findings showed no evidence of obstruction, malignancy, right heart failure, cirrhosis, thrombosis, or infection. She was not taking calcium channel blockers which has been shown to be associated with chylous ascites [8]. The diagnosis of SLE in our patient was made on the basis of 2012 systemic lupus international collaborating clinics (SLICC) criteria [9].

Several mechanisms have been postulated as the cause of chylous ascites in patients with SLE. It is thought that inflammation of lymphatic vessels and cisterns increase the permeability of walls leading to extravasation of chyle. Treatment response to immunosuppressants including corticosteroids supports this theory [7].

There are no standard guidelines for the treatment of chylous ascites. The initial approach is with high protein and low-fat diet with medium chain triglycerides. In the case of SLE associated chylous ascites, immunosuppression with high-dose steroids followed by steroid-sparing therapy has been generally utilized as illustrated in our case. Other possible treatment modalities include multiple therapeutic paracentesis, use of sclerosing agents or suture ligation of thoracic duct and peritoneovenous shunting [3-4].

## Conclusions

This case represents an extremely rare presentation of chylous ascites in SLE. It can be initial manifestation of the disease itself as seen here. Case reports such as these are extremely important to help us better understand this clinical syndrome. We also advocate consideration of SLE in any patient with chylous ascites to ensure timely recognition and treatment of this potentially reversible cause.
